# Effects of a short-term removal of the dominant male on vocalization in captive groups of large-billed crows (*Corvus macrorynchos*)

**DOI:** 10.1098/rsos.241458

**Published:** 2025-04-16

**Authors:** Illia Aota, Mayu Takano, Ei-Ichi Izawa

**Affiliations:** ^1^ Department of Psychology, Keio University, Minato-ku, Tokyo, Japan; ^2^ Tokyo Metropolitan Institute of Medical Science, Setagaya-ku, Japan; ^3^ Japan Society for the Promotion of Science, Chiyoda-ku, Tokyo, Japan

**Keywords:** dominance hierarchy, dominance signal, vocalization, birds, fission–fusion

## Abstract

Dominance hierarchy is widespread among group-living animals as a conflict resolution strategy to avoid the cost and risk of fights among individuals. Dominance signals are well-known mechanisms that allow individuals to assess their opponent’s fighting ability without physical contact, thereby maintaining dominance relationships. In fission–fusion societies, where group composition is fluid, dominance status can shift depending on the current group members. In such situations, vocal signals may be particularly useful as dominance signals due to their easy modification by the signaller. In this study, we investigated the relationship between rank-dependent behaviours and rank ascending by temporarily removing individuals from captive groups of large-billed crows (*Corvus macrorhynchos*). We removed either the first-ranked or third-ranked individuals from the group for 1 day and compared the behaviours of the remaining group members before the removal, during the removal and after the removed individuals rejoined the group. We found that the number of sequential *ka* calls, which is assumed to be a status signal, increased only during the removal of first-ranked individuals and decreased after they rejoined the group. These results suggest that sequential *ka* calls serve as dominance signals, and the subordinates flexibly adjust their vocalization depending on the presence of high-ranked individuals.

## 1. Introduction

Group-living animals often establish dominance hierarchies, a social network with transitive patterns of dominant relationships among individuals [1,2]. These hierarchies provide a conflict resolution mechanism, reducing the risk of injury caused by agonistic conflicts over limited resources [3–5]. Dominance signals and individual recognition are suggested mechanisms for assessing resource competitors [6], facilitating conflict resolution by avoiding the costs associated with overt aggression and injuries [7–10]. These mechanisms are integral to the formation and maintenance of dominance hierarchies.

Dominance signals are specific morphological and behavioural phenotypes often linked to fighting ability, allowing animals to assess their chances of success in conflicts when their dominance relationship is uncertain [10–12]. Individual recognition enables animals to modify their behaviours against an opponent. They avoid conflict, by drawing on past social interactions [13–15] or inferred information about the opponent from aggressive interactions with known individuals [16,17]. These two evaluation mechanisms are not mutually exclusive, although the social structure differences, especially group size and member stability, can influence which one is favoured [18,19]. In societies where groups are large so that individuals often encounter unfamiliar members, the cost of remembering others is high. Hence, dominance signals are thought to play an important role in forming and maintaining dominance hierarchy [11,20].

In birds and mammals, vocal signals are well documented as dominance signals. Acoustic features, such as song-sequence structure, pitch, amplitude or emission rate, correlate with dominance status, physical characteristics, aggressiveness and willingness to escalate, and these signals are suggested to function as dominance signals [21–25]. For example, high-ranking male black-capped chickadees (*Poecile atricapillus*) produce songs more frequently, for longer durations, and begin singing earlier during the dawn chorus compared with lower ranked males within the same flock [26]. Unlike visual signals such as body colour, vocal signals can be turned on and off flexibly in response to changes in social context, allowing individuals to quickly adjust to shifts in their social relationships. This flexibility is especially critical in societies characterized by a high degree of fission–fusion dynamics, where group membership and cohesion are fluid [27]. However, such fluidity does not imply that these groups are merely anonymous aggregates without any social structure or relationships. Instead, they consist of distinct social layers, where both related and unrelated individuals form subgroups or temporary stable units. In such societies, the frequent changes in group composition may limit opportunities for higher ranked, dominant individuals to assert their dominance over lower ranked ones through direct interactions, potentially destabilizing the dominance hierarchy [28]. Vocal signals may play a role in maintaining social order by allowing individuals to assert dominance or adapt to social changes without direct contact.

Chimpanzees (*Pan troglodytes*) and spotted hyenas (*Crocuta crocuta*) exhibit a high degree of fission–fusion dynamics, enabling members to split into smaller subgroups or forage independently over large areas. Despite this spatial flexibility within groups, inter-group interactions are antagonistic, with clear boundaries and confrontations between groups. Vocal signals play a role in enabling individuals to adapt to their socially fluid and spatially dispersed living conditions. High-ranked chimpanzees use *pant-hoots*, long distance calls, to recruit allies and coordinate group movements [29,30]. Similarly, high-ranked hyenas frequently emit *whoops* to recruit group members for territorial defence and group hunting [31]. Observational studies have consistently shown that higher ranked individuals utilize these vocalizations more frequently, indicating their role as advertisements of dominance [29,31]. However, while the relationship between the rank and vocal signals is well documented through observational studies, experimental investigations exploring how vocal behaviour dynamically adjusts in response to short-term rank changes remain limited. By conducting experimental manipulations, such as sequentially removing individuals from species with pronounced fission–fusion dynamics, we can explore how the vocalizations of remaining group members adjust to these absences and determine which ranks significantly influence vocal behaviour.

Corvids are ideal birds for studying the involvement of vocal signals in dominance hierarchies without the effect of rank inheritance. Their social communications depend not only on the visual domain but also on the vocal domain for exchanging information such as sex, identity, group membership, dominance rank and rank reversals of dominance hierarchy [32–38]. Ravens and large-billed crows are corvids that show moderate to high degrees of fission–fusion dynamics, characterized by fluid associations based on foraging opportunities and social interactions rather than fixed territories [39,40]. Non-breeders form temporary groups that split and reassemble daily, often showing site fidelity and forming ‘local’ groups through repeated interactions that support the establishment of dominance hierarchies [41,42]. These social structures also facilitate affiliative relationships, providing advantages such as support during conflicts and opportunities for rank acquisition [43,44]. Previous studies have shown that the most dominant, first-ranked individuals in ravens and large-billed crows emit specific types of calls more frequently than lower ranked individuals [45,46]. Furthermore, an experimental removal study of a captive group of juvenile ravens demonstrated that removal of the first-ranked member for one month resulted in an increase in food-associated calls from the second-ranked member, suggesting sensitivity to changes in group members and an adaptation to a new rank [46].

This study aimed to investigate the effects of a 24 h removal of either the first-ranked or third-ranked group member on the sequential *ka* calls and social behaviours of captive flocks of large-billed crows (*Corvus macrorhynchos*). By focusing on these short-term removals, this study explores how temporal rank changes influence vocal and social behaviours, providing insights into the mechanisms underlying dominance hierarchies in birds. Large-billed crows take 3–4 years to reach sexual maturity, during which time they form groups of non-breeders [47,48]. These sub-adult large-billed crows are known to form and maintain a linear structure of the dominance hierarchy under captive conditions, and once established, the relationships are relatively stable for more than a year [13,49,50]. In this species, dominance is not determined by body size or tarsus length but rather by aggressive behaviour, with more aggressive individuals dominating less aggressive ones [13]. The first-ranked male frequently emits a specific dominance-related vocalization, the sequential *ka* call, which probably serves as a dominance signal [45]. We predicted that the removal of the first-ranked individual would result in an increased frequency of sequential *ka* calls among the remaining group members, particularly from the second-ranked individual, as the absence of the first-ranked member creates a temporary opportunity for rank ascension. Upon the return of the first-ranked individual, we expected a decrease in the frequency of these vocalizations. Additionally, we predicted an increase in aggressive behaviours and allopreening among lower ranked individuals, as previous research has suggested that male allopreening in this species serves as a dominance signal [49]. To further investigate, we also removed the third-ranked individual to determine whether changes in *ka* calls and social behaviours were specific to the absence of the first-ranked individual or a generalized response to the absence of any group member. This manipulation enabled us to assess whether behavioural changes in the second-ranked member were specifically linked to the removal of the first-ranked individual or merely reflective of the group’s response to a missing member. Additionally, the removal of the third-ranked individual allowed us to explore whether the absence of this member triggered behavioural changes in the fourth- and fifth-ranked individuals, whose relative ranks increased in its absence.

## 2. Material and methods

### 2.1. Subjects and animal housing

Subjects were 10 male sub-adults (3 years old) of large-billed crows [48]. The crows were housed in two groups of five birds in two outdoor aviaries (180 m² × 3 m high each). They were captured in Tokyo and neighbouring areas in Japan during their yearling stage after fledging, with authorization from the Japanese Ministry of the Environment (Permission nos. 29030001, 114001, 301482). Their sex was identified by blood DNA [51]. Interior aviaries contained sufficient wooden perches for all the birds to perch simultaneously. Water and food (i.e. dog food, chicken eggs and supplements) were always available in the aviaries.

### 2.2. Dominance rank in pre-experimental period

Before conducting the removal experiment, dominance ranks within each group were determined based on the daily observation data of social interactions collected between April and June 2020. Direct observation was conducted based on 15 min sessions using all-occurrence sampling method [52]. Video observations were carried out using spherical cameras (PIXPRO sp360 4K, Kodak) mounted on the aviary ceilings to capture the entire interior space, including perches and floors. Video recording sessions lasted 60 min each and were analysed offline. All sessions of direct observations and video recording occurred between 08.00 and 16.00. Video recording data were used offline to code social behaviour using the software BORIS 7.10.5. Based on direct observation and video-based data, we collected instances of aggressive behaviour (i.e. jab, peck, kick or displacing approach) and the initiator identity as well as submissive behaviour (i.e. avoid, submissive begging vocalization or retreat) and the recipient identity, in a similar way to what we did in our previous studies [49,50]. In group A, 322 instances of aggressive interactions were recorded based on 10.2 h of data (direct observation: 25 sessions, averaging 1.1 per day; video recording: 4 sessions averaging 2 per day). In group B, 393 instances were documented based on 11.4 h of data (direct observation: 25 sessions, averaging 1.1 per day; video recording: 3 sessions, averaging 2.5 per day). Dominance ranks were determined based on a modified version of David’s score [53] after confirming the significant linearity of hierarchy by calculating linearity index *h'* [54].

### 2.3. Removal–rejoin experiment

To examine how the temporary absence of the first-ranked male affected lower ranked individuals’ behaviours, we conducted an experimental removal–rejoin study. For comparison, the third-ranked male was also removed under similar conditions. The experiment was carried out in both groups (groups A and B). In each group, the first-ranked and third-ranked males were removed individually during separate trials to ensure only one individual was absent at a time.

**Figure 1.**
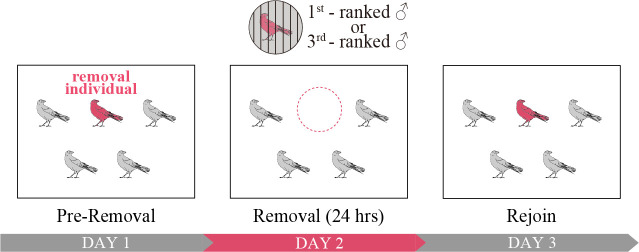
Experimental schedule. Removal individual was separated from the group for 24 h. Behavioural data were collected separately in the three sessions: pre-removal, removal and rejoin.

Each experimental condition consisted of three sessions conducted on consecutive days: pre-removal, removal and rejoin. The pre-removal session was performed a day before the removal and reflected the birds’ usual housing conditions. In the removal session, either the first- or third-ranked male was gently captured using a net and removed from the aviary for 24 h (figure 1). During their absence, the removed individual was housed in an indoor cage (40 × 60 × 40 cm) where it was visually and acoustically isolated from the group. In the rejoin session, the removed individual was returned to the aviary, restoring normal housing conditions. The removal and rejoin experiments were conducted three times across different seasons: July 2021, November 2021 and January 2022. Each trial included a two-week interval between the removal of the first-ranked and third-ranked individuals. The order of removal was randomized and counterbalanced as much as possible between seasons. Note, however, that the third-ranked removal experiment for group A in November 2021 was not conducted due to an external accident at the facility; thus, it was performed twice (in July 2021 and January 2022). Consequently, the number of third-ranked removal sessions is one fewer than the first-ranked removal sessions. Experimental protocol was approved by the Animal Care and Use Committee of Keio University (No. 21018-0).

**Figure 2.**
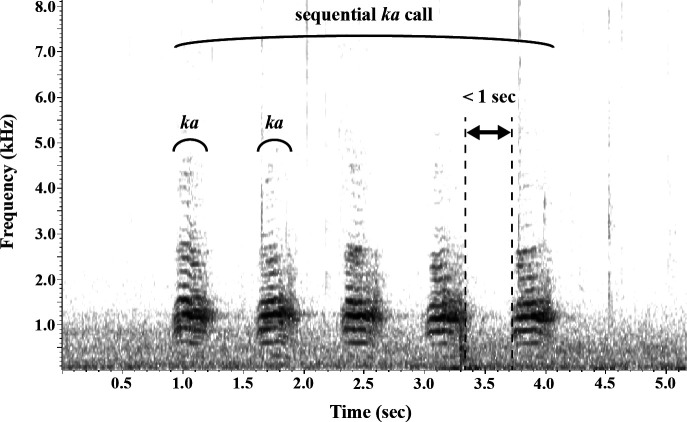
A spectrogram of sequential calls which consisted of repetitive calls within 1 s inter-call interval. Spectrogram was represented with fast Fourier transform window size = 1024, Hanning window, hop size = 5.80 ms (frame overlap = 75%) and frequency grid spacing = 43.1 Hz.

Each session spanned 24 h starting at 9.00. During these sessions, the birds’ behaviours were video recorded for 1 h at three specific times: 9.00, 11.00 and 15.00. Audio recordings of vocalizations were also collected using a pulse code modulation recorder (PCM-D10, SONY) to analyse vocal behaviour. Using the video and audio data, we recorded instances of aggressive behaviours, allopreening bouts and sequential *ka* calls, noting the initiators’ identities. Sequential *ka* calls were defined as separate sequences if an inter-call interval was longer than 1 s (figure 2) using Raven Pro 1.6 software [55].

### 2.4. Statistical analyses

We applied generalized linear mixed models (GLMMs) to examine how the removal of the first-ranked individual affected the behaviour of other group members. Specifically, we evaluated the numbers of sequential *ka* calls, aggressive behaviour and allopreening. For sequential *ka* calls, we used a GLMM with a negative-binomial distribution and log link function, as the data contained many zeros. The dependent variable was the number of sequential *ka* calls, while independent variables included individual rank (treated as a continuous variable from top 1 to bottom 5), removal condition (first-rank removal, third-rank removal and no removal), interaction of rank and removal condition (rank × removal condition) and season condition (July, November and January). The no removal condition, which served as the control, comprised pre-removal and rejoin conditions, as these were consistent with housing conditions. Seasonal effects were included to account for potential variability in behaviour caused by environmental or social factors across different times of the year. January was set to the reference level. To account for repeated measures and the imbalance in number of repetitions, we included individual identity as a random effect. *Post hoc* comparisons were conducted to investigate differences between conditions within specific ranks only when the interaction term was significant, focusing on the second-, fourth- and fifth-ranked individuals. Tukey’s pairwise *post hoc* comparisons were conducted using the *emmeans* package. To address the limitations posed by the relatively small sample size, we performed bootstrapping with 1000 resamples. This method was used to estimate 95% confidence intervals (CI) for the model coefficients and validate the robustness of the fixed effect estimates. The bootstrapping procedure involved resampling the data with replacement and refitting the GLMM to each resample.

For aggressive behaviour and allopreening, we performed GLMMs using a zero-inflated negative-binomial distribution and log link function. This model, which combines a count model and logit model, was chosen because dominance relationships between the initiator and recipient often determine whether the behaviour occurs [13,49]. The count model included the same independent variables as the sequential *ka* call model, with individual identity as a random effect. The logit model incorporated the relative dominance (dominant or subordinate) of the initiator. This framework allowed us to distinguish between structural zeros (due to dominance dynamics) and random zeros (from stochastic processes). When significant interactions were detected, no *post hoc* analyses were conducted, as the primary focus was on the interaction effects themselves. Bootstrapping with 1000 resamples was also performed to estimate 95% CI and validate the robustness of the fixed effect estimates, following the same procedure as for sequential *ka* calls.

Model diagnostics were conducted to ensure the validity of the analysis. Residual diagnostics and overdispersion checks were performed using the *DHARMa* package. Residual diagnostics revealed no significant deviations, and overdispersion tests confirmed that the models fit the data appropriately (electronic supplementary material, S1–S3). Multi-collinearity was assessed with the *performanc*e package, which calculated variance inflation factors for a simplified model without interaction terms. This step ensured that the independent variables (rank, removal condition and season condition) were not highly correlated, as high multi-collinearity could compromise the stability and interpretability of the model coefficients. Results indicated acceptable levels of multi-collinearity for rank and condition. All models were fitted using the mixed model function from the *GLMMadaptive* package using R v. 4.0.5 [56].

## 3. Results

### 3.1. Sequential *ka* calls

A total of 863 and 350 sequential *ka* calls were collected from groups A and B, respectively, throughout the experiment. GLMM analysis revealed a significant effect of first-rank removal (*β* = 5.050, 95% CI [1.525, 8.716], *p* < 0.01; table 1). Seasonal effects were included as a fixed factor, but no significant effects were detected for sequential *ka* calls (July, 95% CI [−1.825, 1.097], *p* = 0.804; November, 95% CI [−0.574, 1.783], *p* = 0.514). Significant interaction was observed between rank × first-rank removal (*β* = −1.159, 95% CI [−2.076, −0.088], *p* < 0.05; table 1) and rank × third-rank removal (*β* = −1.207, 95% CI [−4.093, −0.416], *p* < 0.05; table 1). The effect of rank was estimated at *β* = −0.387 (95% CI [−1.1179, 0.032], *p* < 0.05). However, as the CI overlapped with 0, the effect was not statistically robust.

**Table 1.** Output of the model from generalized linear mixed models for sequential calls.

variables	estimate	s.e.	*z* value	95% CI lower limit	95% CI upper limit	*p*
*intercept*	**2.504**	**0.637**	**3.932**	**0.727**	**4.138**	**<0.01**
*rank*	**−0.387**	**0.188**	**−2.060**	**−1.117**	**0.032**	**<0.05**
*removal condition*						
*first removal*	**5.050**	**1.609**	**3.139**	**1.525**	**8.716**	**<0.01**
*third removal*	−2.173	1.431	−1.519	−0.111	6.616	0.129
*season condition*						
*July*	−0.128	0.515	−0.248	−1.825	1.097	0.804
*November*	0.361	0.554	0.652	−0.574	1.783	0.514
*rank* × *first removal*	**−1.159**	**0.456**	**−2.542**	**−2.076**	**−0.088**	**<0.05**
*rank* × *third removal*	**−1.206**	**0.542**	**−2.2261**	**−4.093**	**−0.416**	**<0.05**

No removal and January were treated as the reference level, respectively. Values in bold indicate statistically significant results (*p* < 0.05).

**Figure 3.**
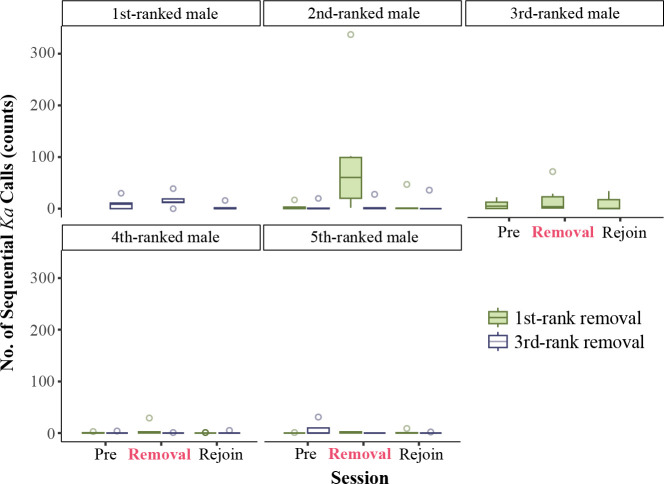
Distribution of sequential *ka* calls across sessions (first-rank removal and third-rank removal) (pre-removal, removal and rejoin) for each rank, represented as box plots. The boxes represent the interquartile range (IQR), with the horizontal line within each box indicating the median. Whiskers extend to 1.5 times the IQR, and outliers are shown as open circles. The removal conditions of the first-ranked and third-ranked individuals are displayed in green and blue, respectively.

**Figure 4.**
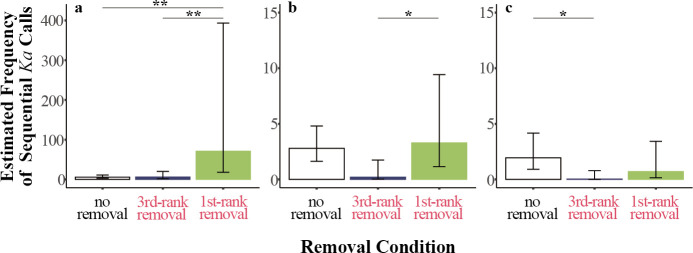
Estimated frequencies of sequential *ka* calls under three conditions (no removal, third-rank removal and first-rank removal) of (a) second-ranked individuals, (b) fourth-ranked individuals and (c) fifth-ranked individuals. Bars represent estimated mean frequencies predicted by the model, and error bars indicate the 95% confidence intervals. Significant differences between conditions are indicated by *p < 0.05, **p < 0.01. Bar colors represent removal conditions, with green indicating first-ranked removal and blue indicating third-ranked removal.

*Post hoc* Tukey tests showed significant differences in the frequency of sequential *ka* calls among conditions across ranks. For second-rank individuals, sequential *ka* calls during first-rank removal were 15.2 times more frequent compared with no removal with no removal calls being only 6.58% as frequent as during first-rank removal (no removal versus first-rank removal, contrast estimate (CE) = −2.731, *p* < 0.01; figures 3 and 4a). Additionally, sequential *ka* calls during first-rank removal were 14.4 times more frequent compared with no removal, with no removal calls being only 6.93% as frequent as during first-rank removal times (third-rank removal versus first-rank removal, CE = −2.670, *p* < 0.01; figures 3 and 4a). There was no significant difference between no removal and third-rank removal conditions (no removal versus third-rank removal, CE = 0.238, *p* < 0.938.). For fourth-rank individuals, sequential *ka* calls during first-rank removal were 21.46 times more frequent compared with third-rank removal, with third-rank removal calls being only 4.66% as frequent as during first-rank removal (third-rank removal versus first-rank removal, CE = −3.066, *p* < 0.05; figures 3 and 4b). There was no significant difference between other pairs of conditions (no removal versus first-rank removal, CE = −0.414, *p* < 0.784; no removal versus third-rank removal, CE = 2.652, *p =* 0.051; figure 3). For fifth-rank individuals, sequential *ka* calls during no removal were 47.42 times more frequent compared with third-rank removal, with third-rank removal calls being only 2.11% as frequent as during third-rank removal (no removal versus third-rank removal, CE = 3.859, *p* < 0.05; figure 3 and 4c). There was no significant difference between other pairs of conditions (no removal versus first-rank removal, CE = 0.744, *p =* 0.709; third-rank removal versus first-rank removal, CE = −3.115, *p* = 0.185).

### 3.2. Aggressive behaviour

A total of 608 and 1137 aggressive behaviours were recorded from groups A and B, respectively, throughout the experiments. GLMM analysis revealed a significant effect of rank, indicating that higher ranked individuals exhibited more aggressive behaviours compared with lower ranked individuals (*β* = −0.698, 95% CI [−0.985, −0.452], *p* < 0.01; table 2). For each one-step increase in numerical rank (e.g. from rank 1 to rank 2), the rate of aggressive behaviour was approximately 0.497 times (50.3% reduction) that of the previous rank. Aggressive behaviour was significantly lower in July compared with January, with the rate of aggressive behaviour decreasing to approximately 0.521 times (48.1% reduction) as high as in January (*β* = −0.653, 95% CI [−0.990, −0.279], *p* < 0.01). The effect of third-rank removal was estimated at *β* = −0.941 (95% CI [−1.764, 0.049], *p* < 0.05). However, as the CI overlapped with 0, the effect was not statistically robust. Similarly, while an interaction between rank and third-rank removal was detected (*β* = 0.356, 95% CI [0.135, 0.689], *p* < 0.05), the CI also overlapped with 0, indicating no statistically significant interaction effect. These findings suggest that rank and season had consistent effects on aggressive behaviour, while the effects of third-rank removal were less certain. No significant effects were observed for other variables (first-rank removal, 95% CI [−0.906, 2.052], *p* = 0.424; November, 95% CI [−0.091, 0.619], *p* = 0.167, rank × first-rank removal, 95% CI [−0.752, 0.335], *p* = 0.496).

**Table 2.** Output of the model from generalized linear mixed models for aggressive behaviour.

variables	estimate	s.e.	*z* value	95% CI lower limit	95% CI upper limit	*p*
negative binomial part						
*intercept*	**3.080**	**0.449**	**6.864**	**2.455**	**3.692**	**<0.01**
*rank*	**−0.698**	**0.169**	**−4.145**	**−0.985**	**−0.452**	**<0.01**
*removal condition*						
*first removal*	−0.613	0.767	0.799	−0.906	2.052	0.424
*third removal*	**−0.941**	**0.444**	**−2.121**	**−1.764**	**0.049**	**<0.05**
*season condition*						
*July*	**−0.653**	**0.189**	**−3.455**	**−0.990**	**−0.279**	**<0.01**
*November*	0.253	0.183	1.381	−0.091	0.619	0.167
*rank* × *first removal*	−0.179	0.264	−0.680	−0.752	0.335	0.496
*rank* × *third removal*	**0.3562**	**0.181**	**1.970**	**−0.135**	**0.689**	**<0.05**
zero inflated part						
intercept	−0.205	0.182	−1.1263			0.260
*dominant (recipient)*	**2.781**	**0.306**	**9.076**			**<0.01**

No removal, January and initiator were treated as the reference level, respectively. Values in bold indicate statistically significant results (*p* < 0.05).

### 3.3. Allopreening

For allopreening, a total of 150 and 56 bouts were recorded from groups A and B, respectively. Allopreening frequency was significantly lower in July compared with January, with the rate of allopreening decreasing to approximately 0.133 times (86.7% reduction) as high as in January (*β* = −2.017, 95% CI [−3.984, −0.653], *p* < 0.01; table 3). No significant effects were observed for other variables, suggesting that the removal of first- and third-ranked individuals had no measurable impact on the allopreening behaviour of the remaining group members (rank, 95% CI [−9.664, 5.681], *p* = 0.510; first-rank removal, 95% CI [−3.892, 8.904], *p* = 0.205; third-rank removal, 95% CI [−10.178, 3.913], *p* = 0.355; November, 95% CI [−1.721, 0.512], *p* = 0.287; rank × first-rank removal, 95% CI [−2.930, 0.926], *p* = 0.135; rank × third-rank removal, 95% CI [−1.509, 2.182], *p* = 0.485).

**Table 3.** Output of the model from generalized linear mixed models for preening bouts.

variables	estimate	s.e.	*z* value	95% CI lower limit	95% CI upper limit	*p*
negative binomial part						
*intercept*	−1.659	1.656	−1.002	−4.663	0.591	0.317
*rank*	−0.280	0.423	−0.662	−0.209	1.014	0.509
*removal condition*						
*1st removal*	2.877	2.272	1.266	−3.892	8.904	0.205
*3rd removal*	−2.207	2.385	−0.9254	−9.664	5.681	0.355
*season condition*						
*July*	**−2.015**	**0.648**	**−3.110**	**−3.984**	**−0.653**	**<0.01**
*November*	−0.591	0.555	−1.064	−1.721	0.512	0.287
*rank* × *first removal*	−1.038	0.695	−1.493	−2.930	0.926	0.135
*rank* × *third removal*	0.433	0.619	0.699	−1.509	2.182	0.485
zero inflated part						
intercept	−0.668	2.5577	−0.261			0.794
*dominant (recipient)*	1.339	1.377	0.9724			0.331

No removal, January and initiator were treated as the reference level, respectively. Values in bold indicate statistically significant results (*p* < 0.05).

## 4. Discussion

This study investigated the short-term effects of removing the first- and third-ranked individuals on the social behaviours and vocalizations of lower ranked members in captive groups of large-billed crows. The findings revealed that the number of sequential *ka* calls correlated with rank, with higher ranked individuals emitting more calls regardless of removal or control conditions, indicating that these calls function as dominance signals. The analysis also identified a significant interaction between rank and removal condition, showing that the second-ranked individual significantly increased its calling rate compared with other lower ranked individuals only in the absence of the first-ranked individual. Unlike the vocal behaviour changes linked to the removal of the first-ranked male, no changes were observed in aggressive or allopreening behaviours during this absence. These findings suggest that sequential *ka* calls are adaptively modulated in response to rank-related opportunities.

Sequential *ka* calls by the second-ranked individual increased exclusively during the absence of the first-ranked male, probably because the second-ranked individual temporarily became the top rank within the group. This suggests that the vocalization is suppressed by the dominant individual and the calls serve to advertise a temporary dominance status. Although previous studies reported a rank-dependent correlation in sequential *ka* call frequency, they only inferred that dominant individuals suppress the vocalizations of lower ranked members [45]. Our study experimentally demonstrates that removing the first-ranked individual enables the second-ranked individual to increase its vocal output, showing that the suppression of lower ranked individuals is actively lifted when rank-related opportunities emerge. This finding underscores the flexible and context-dependent nature of sequential *ka* calls in adapting to social dynamics.

In contrast, removing the third-ranked individual led to a decrease in sequential *ka* calls by the fourth- and fifth-ranked individuals. Although this outcome seems counterintuitive, as the absence of a higher ranked individual should create a rank improvement opportunity, it can be attributed to the social risks linked to dominance-specific vocalizations. In species like ravens, dominance signals can provoke aggression from higher ranked individuals, prompting lower ranked members to adopt conservative strategies to avoid conflict [46]. In this study, the fourth- and fifth-ranked individuals may have suppressed their calls to reduce the risk of drawing attention or aggression from the remaining higher ranked members. This underscores the context-dependent nature of sequential *ka* calls, where individuals weigh the benefits of dominance signalling against the potential costs of social conflict. In this experiment, the first-ranked individual was isolated both visually and acoustically, preventing us from discerning whether their physical presence or vocalizations specifically suppressed the calling behaviour of lower ranked individuals. To address this, future experiments should use playback methods to test whether the vocalizations of the first-ranked individual alone are sufficient to inhibit subordinate vocal output. Such studies could clarify the relative roles of visual and acoustic cues in social suppression and dominance regulation among large-billed crows.

It is important to acknowledge that the observed effect may partly result from the small sample sizes in this study. While the increase in calling frequency by the second-ranked individual was visually apparent, the changes observed in the fourth- and fifth-ranked individuals should be considered preliminary and warrant further investigation. Future studies with larger sample sizes and additional groups are needed to validate these patterns and better understand the conditions under which lower ranked individuals adjust their vocal output.

This study found no clear effect of first-rank removal on aggressive behaviour. The findings on aggressive behaviour differ from those of previous removal studies on cichlids. An experimental study with cichlids showed that removing the dominant males increased aggression among subordinate males, who changed their body colour to reflect dominance within 24 h of the removal [57]. The inconsistency between the results of this study and previous research on cichlids can be explained by another possibility. Given the territorial breeding ecology of the cichlids in the previous study, the housing space (i.e. a tank) is one of the most critical resources for breeding. Occupying a novel territory by removing the most dominant male may drive a rapid change in behavioural and morphological phenotypes immediately after dominance rank turnover [57,58]. On the other hand, as non-breeder crows are non-territorial, the formation of dominance ranks among non-breeders is unlikely for space competition. We cannot exclude the possibility that sexually matured breeder crows may rapidly increase their aggression when taking over new territory, as found in the previous cichlid study. Examining the interplay between species-specific territorial behaviours and developmental stages (e.g. non-breeders versus breeders) would help clarify these differences. Conducting similar removal experiments on sexually mature large-billed crows would provide valuable insights into how ecological and social factors shape dominance-related behaviours.

A previous study on captive groups of large-billed crows reported that male allopreening occurred unidirectionally, from dominant to subordinate individuals, suggesting that male-to-male allopreening functions as a dominance signal to maintain dyadic dominance relationships and, consequently, the dominance hierarchy [49]. Based on this finding, it might be expected that allopreening as a dominance display would increase during the removal of first-ranked males. However, this was not observed. The absence of changes in allopreening during the first-rank removal may stem from the dyad-specific nature and the time-intensive aspect of male allopreening. In another study on large-billed crows [59], consistent male allopreening required approximately two weeks to develop and occurred only in certain dyads. Moreover, compared with the groups observed in the earlier study [49], this study recorded fewer male allopreening interactions, with some dyads showing none. In the groups observed here, allopreening may not serve as a mechanism for reinforcing dominance between males. These group-specific behavioural traits might explain why first-rank removal had no effect on allopreening.

It is also important to consider the potential effects of stress caused by the temporary separation of group members. Stress-induced physiological responses, such as the release of corticosterone and serotonin, are thought to play a critical role in forming and maintaining dominance hierarchies [60]. Stress responses can enhance an individual’s ability to perform dominance displays and retain social memory of losing, thereby stabilizing social ranks [61–63]. It would be interesting to examine whether the higher ranking individuals are stressed, and whether the high-ranked individuals respond to acute stress more than low ranked. However, the absence of increased vocalizations following the removal of the third-ranked individual suggests that this individual may not possess strong social bonds within the group. Furthermore, studies on ravens have shown that individual separation can lead to increased stress levels, particularly in socially well-integrated individuals. In contrast, poorly integrated individuals experience less stress during separation, possibly because group living itself is more stressful for them [64]. It is possible that the observed changes in vocalizations may partly reflect stress responses to social disruption, particularly among individuals with strong social ties, which will also need to be verified in future studies.

Our findings demonstrate that sequential *ka* calls are flexibly adjusted in response to temporary social structure changes and are closely linked to rank-related opportunities. The increased calling by the second-ranked individual highlights the role of these vocalizations in signalling temporary dominance status. Importantly, the lack of apparent changes in aggression and allopreening emphasizes the significance of vocal signals over physical contact in maintaining dominance hierarchies among large-billed crows. Unlike species with rigid hierarchies, large-billed crows prioritize adaptability over strict social cohesion, enabling them to respond effectively to dynamic social and ecological challenges. The use of vocal signals may allow large-billed crows to effectively respond to dynamic social and ecological challenges while maintaining dominance hierarchy. By demonstrating rank-dependent vocalizations in an avian species, this study broadens our understanding of the role of vocal communication in fission–fusion societies and highlights the unique balance of flexibility and structure in avian social behaviour.

## Data Availability

The processed datasets supporting this article have been uploaded as part of the electronic supplementary material [65].

## References

[1] Shizuka D, McDonald DB. 2015 The network motif architecture of dominance hierarchies. J. R. Soc. Interface **12**, 20150080. (10.1098/rsif.2015.0080) 25762649 PMC4387537

[2] Drews C. 1993 The concept and definition of dominance in animal behaviour. Behaviour **125**, 283–313. (10.1163/156853993x00290)

[3] Bernstein IS. 1981 Dominance: the baby and the bathwater. Behav. Brain Sci. **4**, 419–429. (10.1017/s0140525x00009614)

[4] Rowell TE. 1974 The concept of social dominance. Behav. Biol. **11**, 131–154. (10.1016/s0091-6773(74)90289-2) 4367951

[5] Aureli F, de Waal FBM. 2000 Natural conflict resolution. Berkeley, CA: University of California Press.

[6] Tibbetts EA, Pardo-Sanchez J, Weise C. 2022 The establishment and maintenance of dominance hierarchies. Phil. Trans. R. Soc. B **377**, 20200450. (10.1098/rstb.2020.0450) 35000449 PMC8743888

[7] Adamo SA, Hanlon RT. 1996 Do cuttlefish (Cephalopoda) signal their intentions to conspecifics during agonistic encounters? Anim. Behav. **52**, 73–81. (10.1006/anbe.1996.0153)

[8] Barrette C, Vandal D. 1990 Sparring, relative antler size, and assessment in male caribou. Behav. Ecol. Sociobiol. **26**. (10.1007/bf00170894)

[9] Pomerantz O, Baker KC. 2017 Higher levels of submissive behaviors at the onset of the pairing process of rhesus macaques (Macaca mulatta) are associated with lower risk of wounding following introduction. Am. J. Primatol. **79**. (10.1002/ajp.22671) PMC5513769 28431190

[10] Rohwer S. 1975 The social significance of avian winter plumage variability. Evolution **29**, 593–610. (10.1111/j.1558-5646.1975.tb00853.x) 28563094

[11] Rohwer S. 1982 The evolution of reliable and unreliable badges of fighting ability. Am. Zool. **22**, 531–546. (10.1093/icb/22.3.531)

[12] Tibbetts EA, Dale J. 2004 A socially enforced signal of quality in a paper wasp. Nature **432**, 218–222. (10.1038/nature02949) 15538369

[13] Izawa EI, Watanabe S. 2008 Formation of linear dominance relationship in captive jungle crows (Corvus macrorhynchos): implications for individual recognition. Behav. Process. **78**, 44–52. (10.1016/j.beproc.2007.12.010) 18294782

[14] Johnsson JI. 1997 Individual recognition affects aggression and dominance relations in rainbow trout, Oncorhynchus mykiss. Ethology **103**, 267–282. (10.1111/j.1439-0310.1997.tb00017.x)

[15] Whitfield DP. 1986 Plumage variability and territoriality in breeding turnstone Arenaria interpres: status signalling or individual recognition? Anim. Behav. **34**, 1471–1482. (10.1016/s0003-3472(86)80218-4)

[16] Grosenick L, Clement TS, Fernald RD. 2007 Fish can infer social rank by observation alone. Nature **445**, 429–432. (10.1038/nature05511) 17251980

[17] Paz-y-Miño C G, Bond AB, Kamil AC, Balda RP. 2004 Pinyon jays use transitive inference to predict social dominance. Nature **430**, 778–781. (10.1038/nature02723) 15306809

[18] Pagel M, Dawkins MS. 1997 Peck orders and group size in laying hens: ‘futures contracts’ for non-aggression. Behav. Process. **40**, 13–25. (10.1016/S0376-6357(96)00761-9) 24897609

[19] Chaine AS, Shizuka D, Block TA, Zhang L, Lyon BE. 2018 Manipulating badges of status only fools strangers. Ecol. Lett. **21**, 1477–1485. (10.1111/ele.13128) 30043472

[20] Shultz S, Gersick AS. 2016 The evolution of signaling complexity: a comment on Sheehan and Bergman. Behav. Ecol. **27**, 16–17. (10.1093/beheco/arv155)

[21] Kitchen DM, Seyfarth RM, Fischer J, Cheney DL. 2003 Loud calls as indicators of dominance in male baboons (Papio cynocephalus ursinus). Behav. Ecol. Sociobiol. **53**, 374–384. (10.1007/s00265-003-0588-1)

[22] Pfefferle D, Fischer J. 2006 Sounds and size: identification of acoustic variables that reflect body size in hamadryas baboons, Papio hamadryas. Anim. Behav. **72**, 43–51. (10.1016/j.anbehav.2005.08.021)

[23] Reby D, McComb K. 2003 Anatomical constraints generate honesty: acoustic cues to age and weight in the roars of red deer stags. Anim. Behav. **65**, 519–530. (10.1006/anbe.2003.2078)

[24] Searcy WA, Beecher MD. 2009 Song as an aggressive signal in songbirds. Anim. Behav. **78**, 1281–1292. (10.1016/j.anbehav.2009.08.011)

[25] Stoeger AS, Baotic A. 2016 Information content and acoustic structure of male African elephant social rumbles. Sci. Rep. **6**, 27585. (10.1038/srep27585) 27273586 PMC4897791

[26] Otter K, Chruszcz B, Ratcliffe L. 1997 Honest advertisement and song output during the dawn chorus of black-capped chickadees. Behav. Ecol. **8**, 167–173. (10.1093/beheco/8.2.167)

[27] Aureli F *et al*. 2008 Fission‐fusion dynamics: new research frameworks.. Curr. Anthropol. **49**, 627–654. (10.1086/586708)

[28] de Silva S, Schmid V, Wittemyer G. 2017 Fission–fusion processes weaken dominance networks of female Asian elephants in a productive habitat. Behav. Ecol. **28**, 243–252. (10.1093/beheco/arw153)

[29] Mitani JC, Nishida T. 1993 Contexts and social correlates of long-distance calling by male chimpanzees. Anim. Behav. **45**, 735–746. (10.1006/anbe.1993.1088)

[30] Clark AP. 1993 Rank differences in the production of vocalizations by wild chimpanzees as a function of social context. Am. J. Primatol. **31**, 159–179. (10.1002/ajp.1350310302) 31936995

[31] East ML, Hofer H. 1991 Loud calling in a female-dominated mammalian society: II. Behavioural contexts and functions of whooping of spotted hyaenas, Crocuta crocuta. Anim. Behav. **42**, 651–669. (10.1016/s0003-3472(05)80247-7)

[32] Boeckle M, Szipl G, Bugnyar T. 2012 Who wants food? Individual characteristics in raven yells. Anim. Behav. **84**, 1123–1130. (10.1016/j.anbehav.2012.08.011) 23162139 PMC3482666

[33] Hopp SL, Jablonski P, Brown JL. 2001 Recognition of group membership by voice in Mexican jays, Aphelocoma ultramarina. Anim. Behav. **62**, 297–303. (10.1006/anbe.2001.1745)

[34] Kondo N, Izawa E, Watanabe S. 2010 Perceptual mechanism for vocal individual recognition in jungle crows (Corvus macrorhynchos): contact call signature and discrimination. Behaviour **147**, 1051–1072. (10.1163/000579510X505427)

[35] Kondo N, Watanabe S, Izawa EI. 2010 A temporal rule in vocal exchange among large-billed crows Corvus macrorhynchos in Japan. Ornithol. Sci. **9**, 83–91. (10.2326/osj.9.83)

[36] Kondo N, Izawa EI, Watanabe S. 2012 Crows cross-modally recognize group members but not non-group members. Proc. R. Soc. B **279**, 1937–1942. (10.1098/rspb.2011.2419) PMC3311902 22217722

[37] Massen JJM, Pašukonis A, Schmidt J, Bugnyar T. 2014 Ravens notice dominance reversals among conspecifics within and outside their social group. Nat. Commun. **5**, 3679. (10.1038/ncomms4679) 24755739 PMC3997804

[38] Marzluff JM, Walls J, Cornell HN, Withey JC, Craig DP. 2010 Lasting recognition of threatening people by wild American crows. Anim. Behav. **79**, 699–707. (10.1016/j.anbehav.2009.12.022)

[39] Izawa EI. 2011 Social ecology of corvids. Jpn. J. Anim. Psychol. **61**, 55–68. (10.2502/janip.61.1.5)

[40] Boucherie PH, Loretto MC, Massen JJM, Bugnyar T. 2019 What constitutes 'social complexity' and 'social intelligence' in birds? Lessons from ravens. Behav. Ecol. Sociobiol. **73**, 12. (10.1007/s00265-018-2607-2) 30930524 PMC6404394

[41] Boucherie PH, Gallego-Abenza M, Massen JJM, Bugnyar T. 2022 Dominance in a socially dynamic setting: hierarchical structure and conflict dynamics in ravens’ foraging groups. Phil. Trans. R. Soc. B **377**, 20200446. (10.1098/rstb.2020.0446) 35000442 PMC8743890

[42] Loretto MC, Schuster R, Itty C, Marchand P, Genero F, Bugnyar T. 2017 Fission-fusion dynamics over large distances in raven non-breeders. Sci. Rep. **7**, 9. (10.1038/s41598-017-00404-4) 28336913 PMC5428508

[43] Braun A, Bugnyar T. 2012 Social bonds and rank acquisition in raven nonbreeder aggregations. Anim. Behav. **84**, 1507–1515. (10.1016/j.anbehav.2012.09.024) 23264693 PMC3518779

[44] Loretto MC, Fraser ON, Bugnyar T. 2012 Ontogeny of social relations and coalition formation in common ravens (Corvus corax). Int. J. Comp. Psychol. **25**, 180–194. (10.46867/ijcp.2012.25.03.05) 25892846 PMC4398861

[45] Kondo N, Hiraiwa-Hasegawa M. 2015 The influence of social dominance on calling rate in the large-billed crow (Corvus macrorhynchos). J. Ornithol. **156**, 775–782. (10.1007/s10336-015-1191-8)

[46] Heinrich B, Marzluff JM. 1991 Do common ravens yell because they want to attract others? Behav. Ecol. Sociobiol. **28**. (10.1007/bf00172134)

[47] Izawa EI. 2017 Inter-individual communication of large-billed crows: hearing, seeing, and touching. Jpn. J. Anim. Psychol. **67**, 11–18. (10.2502/janip.67.1.2)

[48] Kuroda N. 1981 Observations on a schizochroismic Corvus macrorhynchos, with notes on its territorial life. J. Yamashina Inst. Ornithol. **13**, 215–227. (10.3312/jyio1952.13.215)

[49] Miyazawa E, Seguchi A, Takahashi N, Motai A, Izawa E. 2020 Different patterns of allopreening in the same-sex and opposite-sex interactions of juvenile large-billed crows (Corvus macrorhynchos). Ethology **126**, 195–206. (10.1111/eth.12992)

[50] Ode M, Asaba A, Miyazawa E, Mogi K, Kikusui T, Izawa EI. 2015 Sex-reversed correlation between stress levels and dominance rank in a captive non-breeder flock of crows. Horm. Behav. **73**, 131–134. (10.1016/j.yhbeh.2015.07.012) 26193673

[51] Fridolfsson AK, Ellegren H. 1999 A simple and universal method for molecular sexing of non-ratite birds. J. Avian Biol. **30**, 116. (10.2307/3677252)

[52] Altmann J. 1974 Observational study of behavior: sampling methods. Behaviour **49**, 227–266. (10.1163/156853974x00534) 4597405

[53] de Vries H, Stevens JMG, Vervaecke H. 2006 Measuring and testing the steepness of dominance hierarchies. Anim. Behav. **71**, 585–592. (10.1016/j.anbehav.2005.05.015)

[54] Appleby MC. 1983 The probability of linearity in hierarchies. Anim. Behav. **31**, 600–608. (10.1016/s0003-3472(83)80084-0)

[55] K Lisa Yang Center for Conservation Bioacoustics. 2024 Raven Pro: Interactive Sound Analysis Software [Computer software]. Ithaca, NY: The Cornell Lab of Ornithology. See https://ravensoundsoftware.com/.

[56] Rizopoulos D. 2019 GLMMadaptive: generalized linear mixed models using adaptive Gaussian quadrature. R package version 0.5–1. (10.32614/cran.package.glmmadaptive)

[57] Maruska KP, Fernald RD. 2010 Behavioral and physiological plasticity: rapid changes during social ascent in an African cichlid fish. Horm. Behav. **58**, 230–240. (10.1016/j.yhbeh.2010.03.011) 20303357 PMC2922674

[58] Maruska KP. 2015 Social transitions cause rapid behavioral and neuroendocrine changes. Integr. Comp. Biol. **55**, 294–306. (10.1093/icb/icv057) 26037297 PMC4615796

[59] Seguchi A, Izawa EI. 2024 Vasopressin 1a receptor antagonist disrupts male-male affiliative relationships formed by triadic cohabitation in large-billed crows. eLife. (10.7554/eLife.103406.1)

[60] Korzan WJ, Summers CH. 2021 Evolution of stress responses refine mechanisms of social rank. Neurobiol. Stress **14**, 100328. (10.1016/j.ynstr.2021.100328) 33997153 PMC8105687

[61] Emerson SB, Hess DL. 2001 Glucocorticoids, androgens, testis mass, and the energetics of vocalization in breeding male frogs. Horm. Behav. **39**, 59–69. (10.1006/hbeh.2000.1635) 11161884

[62] Timmer M, Sandi C. 2010 A role for glucocorticoids in the long-term establishment of a social hierarchy. Psychoneuroendocrinology **35**, 1543–1552. (10.1016/j.psyneuen.2010.05.011) 20576360

[63] Weger M, Sevelinges Y, Grosse J, de Suduiraut IG, Zanoletti O, Sandi C. 2018 Increased brain glucocorticoid actions following social defeat in rats facilitates the long-term establishment of social subordination. Physiol. Behav. **186**, 31–36. (10.1016/j.physbeh.2018.01.008) 29331615

[64] Stocker M, Munteanu A, Stöwe M, Schwab C, Palme R, Bugnyar T. 2016 Loner or socializer? Ravens’ adrenocortical response to individual separation depends on social integration. Horm. Behav. **78**, 194–199. (10.1016/j.yhbeh.2015.11.009) 26631484 PMC4754940

[65] Aota I, Takano M, Izawa EI. 2025 Supplementary material from: Effects of a short-term removal of the dominant male on vocalization in captive groups of large-billed crows (Corvus macrorynchos). Figshare. (10.6084/m9.figshare.c.7735211) PMC12000551 40242340

